# Effects of Community Based Fall Prevention Program for Elderly People in Busan Metropolitan City, Korea 

**Published:** 2019-08

**Authors:** Sung-Il Cho, Jong-Gyeong Kim, Chang-Sik Choo, Hye-Yeong Kang, Yeon-Jeong Kim, Joonpil Cho, Jeongyee Bae

**Affiliations:** ^*a*^International Safe Community Research Center of Busan Metropolitan, Republic of Korea.; ^*b*^Public Official, Busan Metropolitan City, Republic of Korea.; ^*c*^Professor, Emergency Medicine of Aju University, Republic of Korea.; ^*d*^International Safe Community Research Center of Busan Metropolitan, Republic of Korea.

## Abstract

**Background:**

Fall related fractures in elderly are one of the most serious health problems in the world. Due to the rapid aging of the elderly in Korea, elderly falls has emerged as a very important social health issue. Falls can result in injuries, a loss of confidence and a subsequent reduction in physical activity and community participant. This work was conducted by the Busan Metropolitan International Safe Community Program in 2018. Purposes The purposes of this study were to develop a community-based fall prevention program and to test the effects of the program on the postural balance and fall efficacy for elderly people.

**Methods:**

36 elderly were recruited from the community in Dong-gu district of Busan Metropolitan City. The program consisted of balance exercises, elastic resistance exercises and prevention education. Postural balance and fall efficacy were evaluated before and at the end of the intervention, using the Functional Reach Test (FRT), Timed Up & Go test (TUG) and the fall efficacy scale-K. The intervention was performed for twice a week in the senior center for 12 weeks. Data were analyzed using paired t-test using the SPSS program.

**Results:**

Developed program was based predominantly on the social cognitive model of behavioral change to successfully increased activities participation in elderly. This program is very useful because it not only focuses on exercise, but participants also learn fall prevention strategies. There were significantly improved in postural balance (FRT; p<.001, TUG; p=0.02) and fall efficacy (p<.001).

**Conclusions:**

This study suggests that this program can improve postural balance and fall efficacy in elders. Therefore, this program is recommended for use in fall prevention programs for elders living in the community.

**Keywords:**

Injury, Aged, Accidental falls, Postural balance

